# Comparative treatment of Sulfasalazine+Ezetimibe combination and Sulfasalazine in a rat model with induced colitis

**DOI:** 10.25122/jml-2023-0194

**Published:** 2023-08

**Authors:** Farrah Rasool Jaafar, Ahmed Abu-Raghif

**Affiliations:** 1 Pharmacology Department, College of Medicine, Al-Nahrain University, Baghdad, Iraq

**Keywords:** anti-inflammatory, adhesion molecule, colitis, Sulfasalazine+Ezetimibe combination

## Abstract

Ulcerative colitis is a chronic inflammatory disease with high mortality and morbidity worldwide. It causes inflammation in the lining of the colon, resulting in several symptoms that negatively impact the quality of life. Unfortunately, there is currently no known cure for this condition. Therefore, it is crucial to explore alternative treatment approaches. This research aimed to investigate the anti-inflammatory and antioxidative effects of a combination therapy involving Sulfasalazine+Ezetimibe compared to Sulfasalazine alone in a rat model of ulcerative colitis. Forty adult rats were divided into four groups for this study. The groups consisted of a control group (negative control), an acetic acid group (positive control), an acetic acid+Sulfasalazine (100 mg/kg per day) group, and an acetic acid+Sulfasalazine (50 mg/kg)+Ezetimibe (5 mg/kg) group. Rats were treated for one week, and colitis was induced by administering 2 ml of 4% (v/v) acetic acid inter-rectally. After sacrifice, the colonic tissue homogenate was analyzed for several markers, including proinflammatory cytokines (TNF-α, IL-1β, NF-κB), oxidative stress markers (malondialdehyde, myeloperoxidase), and adhesive molecule markers (E-selectin, ICAM-1). Sulfasalazine and the combination of Sulfasalazine+Ezetimibe significantly reduced the colonic levels of TNF-α, IL-1β, NF-κB, MDA, and E-selectin in the homogenate. However, the combination therapy of Sulfasalazine and Ezetimibe demonstrated a superior effect.

## INTRODUCTION

Ulcerative colitis is a chronic disease characterized mainly by inflammation and ulcers in the lining of the large intestine, part of the gastric intestinal tract (GIT). This condition can cause a range of symptoms that significantly impact the quality of life. Symptoms may include diarrhea, abdominal pain, and fatigue, leading to malnutrition and weight loss [1, 2]. Furthermore, these symptoms can affect the mental health and social functioning of individuals, thus greatly impacting their overall quality of life.

The etiology of ulcerative colitis remains unknown, but it is believed to involve irregular inflammation in response to antigens, whether of internal, microorganism, or environmental origin, resulting in the propagation and worsening of this inflammation [3, 4]. Several studies have observed abnormal inflammatory responses in various locations of the colon, characterized by an increase in the number of white blood cells, T lymphocytes, mast cells, and other types of inflammatory cells that release inflammatory mediators upon activation [5, 6]. The activation of these cells leads to the secretion of various proinflammatory cytokines and chemotactic substances in improper amounts, causing damage to the surrounding tissues and the spread of the inflammatory response. Some inflammatory cytokines associated with ulcerative colitis include tumor necrosis factor alpha (TNF-α) and interleukin-1 beta (IL-1β) [7].

It is important to note that early treatment of ulcerative colitis can improve outcomes and prevent complications. Treatment can reduce the severity and frequency of symptoms, leading to an improved quality of life. Additionally, it can prevent or delay the need for surgery, which is often associated with complications. Early treatment also reduces the risk of colon cancer, a severe complication of ulcerative colitis. Therefore, early treatment plays a crucial role in managing ulcerative colitis. Despite significant progress in its treatment, the long-term use of certain drugs can lead to drug-induced toxicity and high relapse rates, which limits the effectiveness of established treatments. Therefore, novel strategies for restoring the altered immune response are needed [8, 9].

Sulfasalazine is a medication commonly used to treat inflammatory bowel disease (IBD). It has been found to effectively reduce inflammation in the gut and joints. However, there are also associated side effects with the use of Sulfasalazine. In this study, we will discuss the uses and mechanism of action of Sulfasalazine, as well as the potential side effects and limitations [10]. The efficacy of sulfasalazine in ulcerative colitis is primarily attributed to its active component, 5-ASA, which is responsible for reducing inflammation. The sulphapyridine component of Sulfasalazine acts as a carrier, preventing the absorption of 5-ASA in the small intestine and allowing it to be released in the colon. However, it is important to note that sulfapyridine accounts for many of the side effects associated with Sulfasalazine [11].

There are several controlled-release systems and dosage forms to ensure that 5-ASA reaches the site of inflammation, such as Olsalazine, which is a prodrug disintegrated in the GIT to form Mesalamine, a potent medication for the treatment of ulcerative colitis, available in various forms, including rectal, oral, and enema dosage forms [10]. Medications containing 5-ASA are often considered effective for managing mild to moderate cases of ulcerative colitis despite their unclear action mechanisms, but they appear to have a topical effect [12]. The major mechanism of salicylate is modulating inflammatory mediators derived from the cyclooxygenase and lipoxygenase pathways [10].

On the other hand, Ezetimibe is a lipid-lowering agent primarily used to treat hypercholesterolemia. Its mechanism of action involves the inhibition of intestinal absorption of phytosterols and related sterols, including cholesterol. In addition to lowering cholesterol, Ezetimibe has gained attention in recent years for its putative anti-inflammatory properties. Studies have shown that Ezetimibe can reduce inflammation in experimental models of atherosclerosis and rheumatoid arthritis. This has sparked interest in exploring its therapeutic potential in other inflammatory conditions, including IBD [11].

Combination therapy, involving the simultaneous use of two or more drugs with complementary mechanisms of action, has gained attention in the field of IBD treatment. The rationale behind combination therapy is to enhance efficacy, minimize side effects, and potentially target multiple pathways involved in the pathogenesis of the disease [12].

In light of the promising individual effects of Sulfasalazine and Ezetimibe, we hypothesized that their combination might result in the best therapeutic effects in colitis management. Therefore, this study aimed to investigate the comparative treatment effects of sulfasalazine alone and the combination of Sulfasalazine and Ezetimibe in a rat model with induced colitis.

Ezetimibe is one of the azetidinone group cholesterol absorption inhibitors and is commonly used as a supplementary treatment to dietary modifications and statin therapy for individuals with hypercholesterolemia [13]. The aforementioned molecular composition corresponds to the chemical formula C24H21F2NO3 [14]. Cholesterol is not absorbed because a transport protein (NPC1L1) in the duodenal brush boundary is blocked. In addition, it inhibits cholesterol excretion in the bile and possesses anti-inflammatory properties [15, 16], as evidenced by the attenuation of articular cartilage alterations mediated by decreases in the levels of inflammatory cytokines [17].

This study aimed to investigate the comparative treatment effects of a combination of Sulfasalazine and Ezetimibe versus Sulfasalazine alone in a rat model with induced colitis. Sulfasalazine is a commonly used medication for treating IBD, while Ezetimibe is an agent primarily used for lowering cholesterol levels. The rationale behind combining these two drugs was to explore their potential effects in ameliorating colitis.

## MATERIAL AND METHODS

### Experimental animals

In our research, adult male albino rats weighing 200–220 g were employed. The rats were housed in polypropylene clean cages in a relatively stable condition, with a temperature of about 24–25 degrees Celsius, humidity of 35–60 percent, and a 12/12 h (day to night cycle). We stopped rat feeding about 24 hours before the induction of colitis to ensure deprivation and complete induction of colitis, but they received water only. The rats were housed in enclosures featuring broad wire mesh flooring as a preventative measure against coprophagy. Before inducing colitis, the rats were deprived of water for 2 hours [18].

### Induction of ulcerative colitis

Rats underwent a fasting period of at least 24 h prior to the induction of colitis. This procedure was performed to ensure proper induction by clearing the colon of fecal matter. Nevertheless, the rats had free access to tap water during this period. The induction of colonic ulceration was conducted experimentally with a modified version of the procedure recommended by Mousavizadeh *et al*. [19]. In this study, rats were administered a single intrarectal infusion of a 4% acetic acid solution at a 5 ml/kg dose for 30 s. The infusion was delivered 8 cm into the colon with a flexible plastic tube while the rats underwent light ether anesthesia (2 mm extrinsic diameter). The release of acetic acid was inhibited by positioning the rats horizontally for 2 min.

### Experimental design

The experiments were divided into 4 categories (ten rats for each group). Group I was non-treated and was the negative control (normal saline infusion through the rectal route). In the other groups, rectal administration of 4% acetic acid (v/v) induced colitis. Group II represented positive control and received normal saline orally, group III was treated orally with Sulfasalazine (100 mg/kg), and Group IV was treated orally with a combination of Sulfasalazine (50 mg/kg) and Ezetimibe (5 mg/kg). In all groups, treatment lasted for 7 days. The duration of treatment was determined according to prior studies on experimental colitis [20, 21].

### Preparation of drugs

The solutions of Sulfasalazine and the Sulfasalazine+Ezetimibe mixture were prepared prior to administration. These drugs were suspended in distilled water. The recommended dosage of Sulfasalazine was 100 mg/kg [22].

### Tissue samples

Upon the completion of the experiment, the rats were euthanized with an excessive dose of diethyl ether. The colon was expeditiously excised subsequent to abdominal dissection. A longitudinal incision was made on the colon, delicately irrigated with normal saline. Colon samples were collected, rinsed with PBS (0.02 mol/L; pH 7.2–7.4), and weighed at a temperature of -80 °C. The tissue samples were sliced into small fragments and subsequently homogenized in a specified quantity of PBS (1 g of tissue in 9 ml of PBS) with a homogenizer. Subsequently, the homogenate was centrifugated at 5000 commotions per minute for 15 min [23]. We measured colon tissue markers, including oxidative stress indicators (MDA and MPO), inflammatory cytokines (TNF-α, IL-1β, and NF-κB), and adhesion molecules (ICAM-1 and E-selectin), using ELISA kits as per the manufacturer’s instructions (Elk Biotechnology - Chania).

### Statistical analysis

Data were summarized, analyzed, and presented using the Statistical Package for Social Sciences (SPSS) version 23. We used the mean and standard deviation to express quantitative variables numerically. One-way ANOVA was used to examine differences in the means of quantitative variables across groups, followed by the post-hoc least significant difference test for evaluating differences in the means of quantitative variables within groups. A p-value of <0.05 indicated significance [24].

## RESULTS

The levels of TNF-α, IL-1β, and NF-κB in the colon tissue homogenate were significantly increased in the colitis group (434.5±53.47 pg/ml, 646.1±58.65 pg/ml and 9.05±0.74 pg/ml, respectively) compared to the healthy control group (139.8±76.57 pg/ml, 142.5±42.09 pg/ml and 1.21±0.75 pg/ml, respectively). The Sulfasalazine+Ezetimibe combination had a statistically significant and higher effect in reducing the levels of TNF-α (147.7±31.12 pg/ml), IL-1β (162.4±28.19 pg/ml) and NF-κB (1.3±0.82 pg/ml) compared to Sulfasalazine (197.25±64.97 pg/ml,190.87±36.86 pg/ml and 2.11±0.88 pg/ml, respectively), as shown in Table 1.

**Table 1:** Proinflammatory cytokines of tissue-level homogenates in different groups, adhesion molecules

Variables	Healthy control (n=10)	Induced colitis (n=10)	Sulfasalazine (n=10)	Sulfasalazine l+Ezetimibe (n=10)	p-value
**Colonic TNF-α** **lpg./m**l	139.8±76.57 lA	434.5±53.47 lB	197.25±64.97 lC	147.7±31.12 lA	**<0.001**
**Colonic IL-1β pg/ml**	142.5±42.09 lA	646.1±58.65 lB	190.87±36.86 lC	162.4±28.19 lA	**<0.001**
**Colonic NF-κB** **lpg/m**l	1.21±0.75 lA	9.05±0.74 lB	2.11±0.88 lC	1.3±0.82 lA	**<0.001**

TNF-α, tumor necrosis factor alpha; IL-1β, interleukin 1-beta; NF-κB, nuclear factor-kappa beta.

The values were expressed as mean ± standard deviation (SD).

As displayed in Table 2 the levels of ICAM-1 and E-selectin were elevated after induction by acetic acid (7.43±0.53 ng/ml and 432.5±71.3 pg/ml, respectively) compared to the healthy control group (1.33±0.58 ng/ml and 165.70±65.12 pg./ml, respectively). However, the Sulfasalazine+Ezetimibe combination produced a better result than Sulfasalazine in reducing ICAM-1 (1.41±0.51 ng/ml and 2.01±0.67 ng/ml, respectively), but no significant difference was observed. The combination had a better result than E-selectin (174.19±53.15 pg/ml and 194.37±50.60 pg/ml, respectively), and the difference was significant.

**Table 2:** Adhesion molecules of tissue-level homogenates in different groups

Variables	Healthy control (n=10)	Induced colitis l(n=10)	Sulfasalazine l(n=10)	Sulfasalazine+Ezetimibe l(n=10)	p-value
**Colonic ICAM ng/ml**	1.33±0.58 lA	7.43±0.53 lB	2.01±0.67 lA	1.41±0.51 lA	**<0.001**
**Colonic E-selectin pg./m**l	165.70±65.12 l A	432.5±71.3 lB	194.37±50.60 lC	174.19±53.15 lA	**<0.001**

Different letters indicate significant differences. ICAM-1, intercellular adhesion molecule-1;

E-selectin, endothelial selectin. Values were expressed as mean ± SD.

For tissue level homogenate oxidative stress markers (MDA and MPO), there were highly significant increases in the colitis group (7.47±1.21 ng/ml and 250.4±30.27 pg/ml, respectively) compared to the healthy control group (1.37±0.39 ng/ml and 105.70±19.70 pg/ml, respectively). The combination of Sulfasalazine+Ezetimibe was more effective in reducing MDA levels (117.7±16.22 pg/ml) than the Sulfasalazine group (137.56±24.69 pg/ml). The homogenate level of MPO in the Sulfasalazine+Ezetimibe group and the Sulfasalazine group was 1.60±0.78 ng/ml and 1.80±0.62 ng/ml, respectively (Table 3).

**Table 3:** Oxidative stress markers of tissue-level homogenates in different groups

Variables	Healthy control (n=10)	Induced colitis (n=10)	Sulfasalazine (n=10)	Sulfasalazine +Ezetimibe l(n=10)	p-value
**Colonic MPO ng/ml**	1.37±0.39 lA	7.47±1.21 lB	1.80±0.62 lA	1.60±0.78 lA	**<0.001**
**Colonic MDA pg/ml**	105.70±19.70 lA	250.4±30.27 lB	137.56±24.69 lC	117.7±16.22 lA	**<0.001**

MPO, myeloperoxidase; MDA, malondialdehyde. The expression of values means ± standard deviation (SD).

Different letters indicate significant differences.

## DISCUSSION

This research showed that Sulfasalazine+Ezetimibe combination significantly reduced the tissue homogenate levels of NF-κB, TNF-α, IL-1β, oxidative markers (e.g., MAD), and adhesion molecules (e.g., E selectin). Unfortunately, no previous studies investigated the effects of the Sulfasalazine+Ezetimibe combination on colitis. A previous study showed that Ezetimibe exerts pharmacological, anti-inflammatory, and immunomodulatory effects, reduces cytokine production, inhibits macrophages, and reduces the levels of circulating inflammatory cytokines. Meanwhile, the mechanism by which sulfasalazine improves inflammatory response in colitis is merely attributed to its anti-inflammatory and antioxidant effects [25, 26]. This effect of Ezetimibe is related to its anti-inflammatory and immunomodulatory activity. It reduces cytokine production, inhibits macrophage aggregation, and reduces circulating inflammatory cytokines. Furthermore, Ezetimibe treatment in mice is associated with a decline in the average number of CD3+ and CD4+ T cells and CD3+, CD4+, and CD45RO+ T memory cells and in the regulation of the JAK/STAT pathway, which is responsible for synthesizing inflammatory cytokines [27-31]. In addition to its effectiveness in reducing the inflammatory, oxidative, and adhesive features of colitis, this combination therapy offers two significant benefits. First, it can mitigate the adverse effects associated with Sulfasalazine. Sulfasalazine monotherapy is associated with many serious side effects, the most important of which is renal injury, which has been extensively reported [32-35]. Secondly, this combination approach can disrupt two concomitant disorders simultaneously (treatment of ulcerative colitis and prevention or treatment of dyslipidemia). IBD is closely linked with several inflammatory mediators and insulin resistance, all potential predisposing factors for atherosclerosis [36, 37].

Furthermore, a study by Visschers *et al*. (2009) primarily utilized Ezetimibe as a lipid-lowering agent, and according to the result of the present study, it has anti-inflammatory activity and lower adverse effects than Sulfasalazine [38]. Hence, Sulfasalazine+Ezetimibe combinations are a good alternative to monotherapy using either Ezetimibe or Sulfasalazine.

## CONCLUSION

In conclusion, the combination of Sulfasalazine and Ezetimibe showed superior therapeutic efficacy in the rat model of induced colitis compared to Sulfasalazine monotherapy. These findings suggest that the dual targeting of inflammation and lipid metabolism may hold promise as a potential therapeutic approach for managing IBD. Further studies are warranted to elucidate the underlying mechanisms and validate these findings in human clinical trials. The combination significantly reduced the levels of pro-inflammatory cytokines TNF-α, IL-1β, and NF-κB, and adhesion molecule E-selectin. The levels of adhesion molecule ICAM-1 and oxidative markers MPO were also reduced, but the difference was not significant. These findings suggest that the Sulfasalazine and Ezetimibe combination could be a promising therapeutic strategy.
